# Comparative Analysis of Carbon, Ecological, and Water Footprints of Polypropylene-Based Composites Filled with Cotton, Jute and Kenaf Fibers

**DOI:** 10.3390/ma13163541

**Published:** 2020-08-11

**Authors:** Jerzy Korol, Aleksander Hejna, Dorota Burchart-Korol, Jan Wachowicz

**Affiliations:** 1 Department of Material Engineering, Central Mining Institute, Pl. Gwarków 1, 40-166 Katowice, Poland; jwachowicz@gig.eu; 2 Department of Polymer Technology, Gdansk University of Technology, Narutowicza 11/12, 80-233 Gdansk, Poland; aleksander.hejna@pg.edu.pl; 3 Faculty of Transport and Aviation Engineering, Silesian University of Technology, Krasińskiego 8, 40-019 Katowice, Poland

**Keywords:** water footprint, carbon footprint, ecological footprint, biocomposites, natural fibers

## Abstract

Composites containing natural fibers are considered environmentally friendly materials which is related to the reduced use of fossil fuels and the emission of carbon dioxide compared to petroleum-based polymers. Nevertheless, a complete evaluation of their environmental impact requires a broader view. This paper presents a carbon, ecological, and water footprints assessment of polypropylene-based composites filled with cotton, jute, and kenaf fibers based on a standardized European pallet (EUR-pallet) case study. Obtained results were compared with unmodified polypropylene and composite with glass fibers. Incorporation of 30 wt% of cotton, jute, and kenaf fibers into a polypropylene matrix reduced its carbon footprint by 3%, 18%, and 18%, respectively. Regarding the ecological footprint, an 8.2% and 9.4% reduction for jute and kenaf fibers were noted, while for cotton fibers, its value increased by 52%. For these footprints, the use of jute and kenaf fibers was more beneficial than glass fibers. Nevertheless, the application of natural fibers caused a 286%, 758%, and 891% drastic increase of water footprint of the final product, which was mainly affected by cultivation and irrigation of crops. Therefore, in a holistic view, the incorporation of natural fibers into the polypropylene matrix definitely cannot be impartially considered as an environmentally friendly solution.

## 1. Introduction

Currently, one of the main trends in research associated with polymer technology is looking for a reduction in the environmental impacts of plastics. Such a trend is expressed by different activities related to the industrial practice, but also the direction of research works. One of the most popular approaches to this problem is the recycling of polymeric waste, the popularity of which is continuously increasing, not only in research works but also in the industry [1,2,3]. Other approaches focus on the incorporation of waste materials from various industries into plastics production technologies. Among commonly investigated solutions can be mentioned the utilization of waste rubber in the manufacturing of polymer composites [4,5], production of polyols for polyurethanes from waste oils [6] or crude glycerol [7,8], or liquefaction of biomass resulting in intermediate compounds for the synthesis of different polymers [9]. Plastics can also be produced from bio-based raw materials. We can mention biopolymers obtained by living organisms, such as poly(lactic acid), poly-3-hydroxybutyrate, or starch, which can be quickly processed into thermoplastic starch [10]. Another way of processing bio-based raw materials into plastics is their incorporation into polymer matrices as fillers, resulting in the generation of materials often referred to as natural fiber composites (NFCs) or wood polymer composites (WPCs) [11,12]. Various lignocellulosic fibers and fillers may be introduced into polymer matrices, either as by-products from different processes or primary materials. The most commonly applied lignocellulosic by-product is wood flour, wood dust, or different types of pulp [13]. Among primary materials, the most popular are different types of fibers, e.g., kenaf fibers [14,15], jute fibers [16,17], or cotton fibers [18,19].

Generally, according to the common understanding, the introduction of bio-based raw materials into polymer matrices is considered environmentally friendly. However, there is still a limited number of works focused on assessing the environmental impact of NFCs and WPCs. The most popular topic is the life cycle assessment (LCA) of these materials [20,21]. In our previous work [22], we combined the LCA with the eco-efficiency analysis of polypropylene composites filled with various natural fibers. Obtained results revealed that the assessment of the environmental impact of these composites is not easy and straightforward. For a complete evaluation, comparative analysis of multiple categories and factors has to be performed, because the results may be very diverse. The simple introduction of natural fibers into a petrochemical-based polymer matrix does not make the material environmentally friendly. It may enhance some ecological aspects of the material, e.g., reduce its global warming potential or ozone depletion; however, the overall effect does not have to be so obvious. Among others, such an effect is related to significant water usage during the cultivation of various crops [23]. Similar observations were made by other researchers [20,24]. Álvarez-Chávez et al. [25] indicated that bio-based substitutes of conventional plastics show some drawbacks despite their potential for the reduction of fossil fuels use and for avoiding the non-degradable bulky plastic waste. The authors pointed out the significant land use, water use, application of hazardous pesticides and fertilizers, as well as transgenic plants and genetically modified seeds, whose long-term environmental and health effects are unknown nowadays.

Therefore, to fully determine the environmental friendliness of WPCs and NFCs, it is essential to analyze the whole spectrum of effects. Such an approach was proposed by Galli et al. [26], who introduced the term of Footprint Family, which integrates the carbon, ecological, and water footprints. These indicators are often not comparable and are based on different approaches to the issue. However together, they may provide a more comprehensive look at the problem of product or process [27]. In his work, Hoekstra [28] shows that ecological and water footprints are providing different pieces of information, so they should be seen as complementary indicators since land can be considered critical in one case, while water use in other. The combination of multiple footprints may provide a synergistic effect, strengthening the advantages and overcoming the drawbacks of particular indicators as presented in Table 1.

Having in mind the factors mentioned above and the environmental trends focused on the enhancement of ecological aspects of plastics production, we adopted the comprehensive approach of Galli et al. [26] in the evaluation of the environmental impact of polypropylene-based natural fiber composites. The presented study was aimed at calculating and evaluating the carbon, ecological, and water footprints of polypropylene and assessing their changes after partial substitution of polymer with kenaf, jute, and cotton fibers. We believe that such research works are very helpful in setting the trends in polymer and plastics technology and in answering the following question related to the health of the planet—Can the substitution of petroleum-based plastics with bio-based materials result in the manufacturing of engineering-useful materials and simultaneously reduce their environmental impact?

## 2. Experimental

### 2.1. Goals and Scope of Analysis

The presented research work was aimed at analyzing and evaluating the environmental footprints of polypropylene-based composites filled with natural fibers which already are and can be used to a greater extent as partial substitutes for virgin polymers. Moreover, to relate the use of natural fibers to more traditional and conventional approaches, analyzed biocomposites and their environmental footprints were compared to the use of neat polypropylene and composites containing glass fibers.

**Figure 1:**
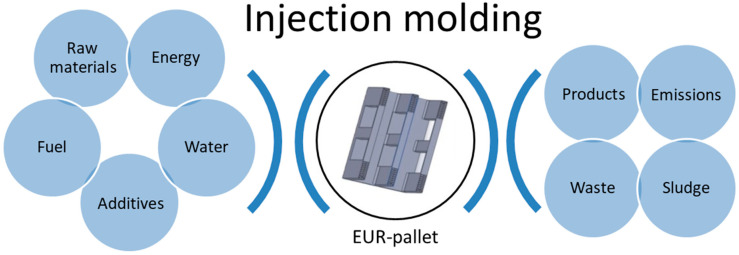
Scope of performed assessment of environmental footprints.

The assessment of carbon, ecological, and water footprints was conducted from the raw materials stage, which takes into account their acquisition and production, to the final product obtained by injection molding technology. Such an approach is often related to cradle-to-gate assessment and does not include the use and recycling of investigated products. The applied approach is schematically presented in Figure 1.

**Figure 2:**
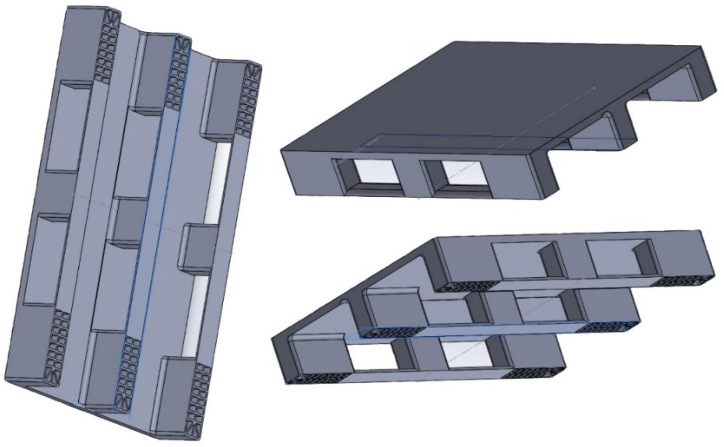
The appearance of standard EUR-pallet..

For analysis, one standard European pallet (EUR-pallet) was selected as the functional unit (FU), according to the European Pallet Association standards. It is closed-deck medium duty EUR-pallet, offered by various companies, e.g., Logistic Packaging (Bucharest, Romania) or Associated Pallets Ltd. & Plastic Pallets UK (Southampton, Great Britain) [29,30]. A schematically analyzed pallet is shown in Figure 2. The mass of the analyzed pallet is 15 kg.

In the presented research work, we applied a plastic pallet as a functional unit, because it is a product commonly applied in various branches of industry all over the world. They are more and more often replacing conventional wooden pallets. According to the “Plastic Pallets Market by Material and Geography—Global Forecast & Analysis 2020–2024” report, the compound annual growth rate for plastic pallets is around 8.0%, while for the total pallet market is around 4.8%, indicating the growing popularity of plastic pallets [31]. Such an effect is related to their performance and longer estimated life (over 40% higher durability), but also convenience associated with their use, i.e., ease of cleaning, lower probability of getting hurt (splinters in non-sanded wood), inconsistent size, and other issues which makes the automatization of the production processes difficult [32,33,34]. Moreover, the multiplicity of plastics and a wide range of their potential properties enables the selection of raw materials considering the desired application of the pallet. Currently, plastic pallets are mainly produced from polypropylene (PP) which is associated with a beneficial combination of its relatively low price and satisfactory (as for this application) mechanical properties. Polypropylene is generally well-analyzed material, and its multiple modifications were developed, including its partial replacement by incorporation of fillers of different origins [35]. Depending on the composition, structure, size, and aspect ratio of fillers, their incorporation may result in lower material costs and the enhancement of its performance. Conventionally, commonly applied are glass fibers [36]. However, researchers are looking for natural and renewable substitutes over the last years, considering pro-ecological trends in the plastics industry [37]. An essential feature of PP is its hydrophobic character, so the incorporation of natural fillers, which are prone to biodegradation, does not result in biodegradable character of PP-based composite [38]. Therefore, it is possible to introduce natural-based, primary, or even waste materials as potential fillers for composites based on the PP matrix. As mentioned in the introduction, the application of bio-based raw materials in polymer technology is considered as very environmentally friendly due to the reduction of the use of fossil fuels and lower emissions of carbon dioxide. For the presented analysis, the following materials were selected as fillers for PP-based composites: cotton fibers (CFs), jute fibers (JFs), and kenaf fibers (KFs) as well as glass fibers (GFs) mentioned above, which are one of the oldest reinforcement used in the manufacturing of polymer composites and were included for comparison. The content of natural fibers in analyzed composites was fixed at 30 wt%. Incorporation of the excessive amount of biodegradable filler may result in biodispersible composite. Such an effect can be noted, when the content of polymer phase is too low and it is not completely covering the filler particles. As a result, the external factors may induce the decomposition of biodegradable filler and loss of the composite’s cohesion. According to the literature, 30 wt% content of biodegradable filler guarantees that the material should not be biodispersible [39]. Moreover, such loading results in the enhancement of mechanical performance which may result, for example, in the lower thickness and smaller weight of the final product. Based on the literature data [35], incorporation of 30 wt% of natural fibers into the PP matrix may result in the increase of tensile strength from 25–33 to 40–45 MPa and the rise of Young’s modulus from 1000—1400 to 3000—3500 MPa. For comparison with material solutions based on natural fibers, a conventional variant with 10 wt% addition of commonly applied glass fibers, which results in the similar mechanical performance of composite, was also analyzed.

Table 2 shows the analyzed formulations of raw materials for the manufacturing of EUR-pallets.

### 2.2. The Methodology of Environmental Footprints Calculation

**Figure 3:**
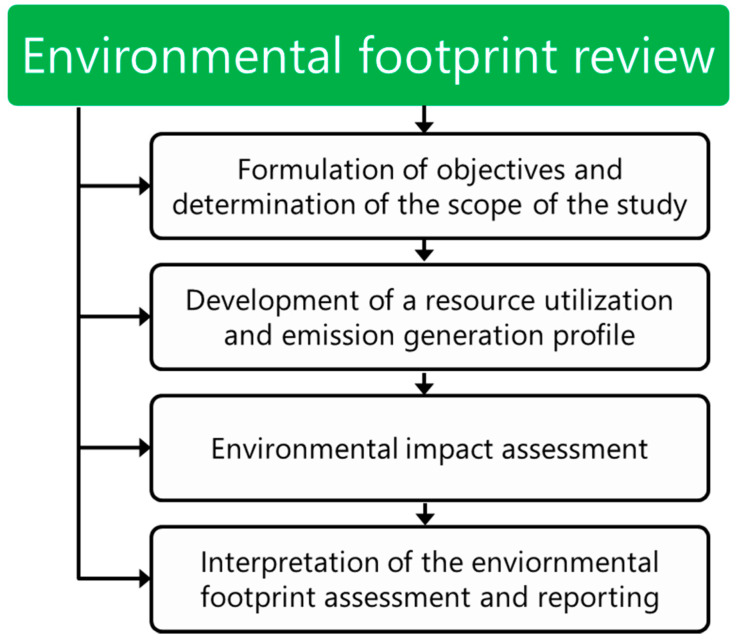
General scheme of environmental footprint assessment.

Some basic principles related to the evaluation of environmental footprints of various products, processes, and organizations, are presented in the European Commission Recommendation 2013/179/EU on the use of standard methods to measure and communicate the life cycle environmental performance of products and organizations [40]. In Figure 3, there is schematically presented the course of the environmental footprint assessment.

**Figure 4:**
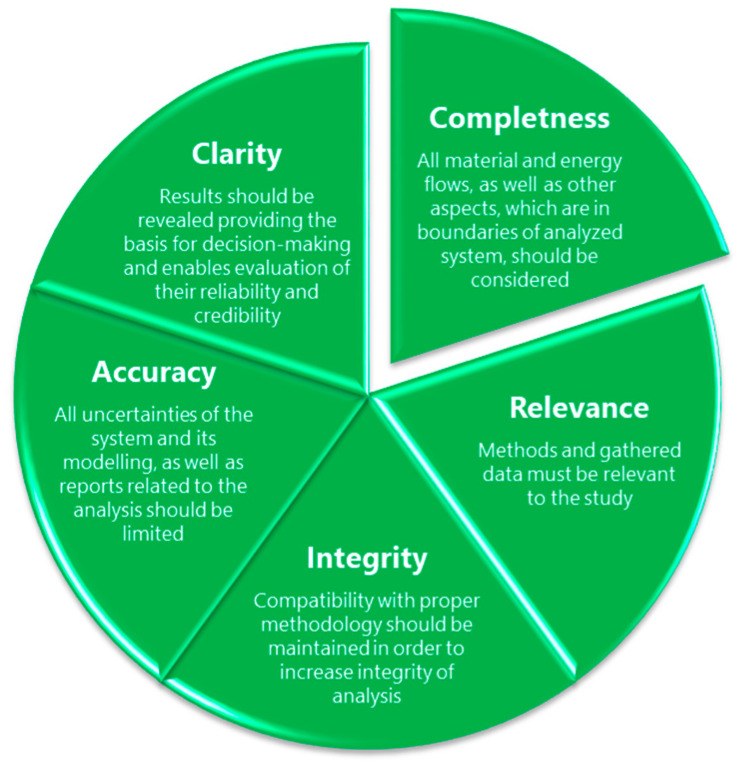
Principles of the environmental footprint assessment.

Moreover, in addition to applying the recommended proper methodology for assessing the individual environmental footprints and taking into account the physicochemical properties of the analyzed materials, assessment requires certain principles be taken into account [40]. These principles were developed to guarantee consistent, reliable, and reproducible assessments of environmental footprints. They aim to provide guidelines for analysis, similarly, as various standards related to different measuring methods, e.g., International Organization for Standardization (ISO), American Society for Testing and Materials (ASTM), or Deutsches Institut für Normung (DIN) standards. To do so, they must be taken into account during every step of environmental footprint study, from formulating the objectives of the study and determining its scope through data collection, environmental impact assessment, and reporting, to verify the assessment results. Schematically, they are presented in Figure 4.

An assessment of the carbon footprint of the analyzed materials was carried out following the method developed by the Intergovernmental Panel on Climate Change (IPCC), currently, one of the most widely propagated methods of calculating the carbon footprint, useful especially at product and technology levels. It allows the determination of the impact of products and technologies, taking into account not only CO_2_ but also other gasses’ emissions. The result is expressed in the form of CO_2_ equivalent—kg CO_2_eq (IPCC 2007) [41] which is calculated by multiplying the actual gas mass by the global warming potential factor (GWP) for a specific gas, making the global warming effects of different greenhouse gases (GHGs) comparable and additive. Table 3 presents potential greenhouse indicators for selected GHGs with different greenhouse potential relative to carbon dioxide. These indicators reflect the extent to which one kilogram of these substances contributes to the greenhouse effect over 100 years. The IPCC method allows the assessment of a single environmental effect which is the greenhouse effect [42]. The magnitude of the greenhouse effect, due to the differences in durability in the atmosphere of different GHGs, varies depending on the time scale. Short-term (20 years), medium (100 years), and long-term (500 years) effects can be considered. In this paper, a 100-year perspective was taken for calculations. As part of the carbon footprint assessment, the IPCC method calculates greenhouse gas emissions by:

- inventory of emissions of all greenhouse gases in the life cycle of the production system;
- converting them into equivalent carbon dioxide (kg CO_2_eq), using global warming potential indicators (GWP), developed by the Intergovernmental Panel on Climate Change;
- adding up the values obtained for calculating the cumulative greenhouse gas emissions.

Guidelines and requirements for design, development, management, reporting, and verification related to the company’s GHG inventory have been described in the ISO/TS 14,067 standard, including the carbon footprint calculation proposal [43]. It was developed because of the need to designate clear, uniform ones and universal rules for determining the carbon footprint, as well as for reporting guidelines and making the results of these calculations publicly available. The authors of the standard emphasize that it is used to assess the carbon footprint of a product as one of the environmental aspects. For this reason, it cannot be used to determine the economic or social consequences associated with the environmental performance of a product or service. An important fact is that according to ISO/TS 14067:2013, the process of calculating the carbon footprint—as in publicly available specification (PAS) 2050—should take into account the idea of the life cycle. Therefore, the greenhouse gas emissions resulting not only from the company’s direct activity are being analyzed. Indirect emissions are also included in the analyses. This standard divides inventory data into two general categories:

- direct GHGs emissions—covering emissions occurring on the enterprise’s premises determined through monitoring, stoichiometric or mass balance; “Direct” in this case means controlled by the enterprise, but this can also be understood as having no prior or later technological history;
- data covering the entries and exits of materials entering and leaving the organization that has their technological history resulting in greenhouse gas emissions; with this breakdown in the standard, greenhouse gas emissions are grouped at three levels:
  1. Emissions from greenhouse gas sources owned or supervised by the enterprise (direct emissions);
  2. Greenhouse gas emissions when generating electricity, heat or steam consumed by an enterprise (indirect greenhouse gas emissions);
  3. Emissions other than indirect energy greenhouse gas emissions, which are the result of the business, but arise in installations that are owned or supervised by other companies.

This means that not only own direct emissions are taken into account for calculations, but also those occurring in the supply chain, which means that the data analysis process itself is time-consuming, labor-intensive, and requires specialized expert knowledge. In many cases, it is computer-aided using specialized software. Also, before starting the analysis, the appropriate functional unit should be determined, and the limits and scope for which carbon footprint will be calculated should be determined. It requires getting to know the entire production process or the overall way the company functions. The analysis covers the following ranges:

- from the cradle to the grave—all stages from the extraction of raw materials to disposal are taken into account; or
- from the cradle to the gate—where the stages from the extraction of raw materials to delivery of the finished product to the customer are counted, including the process of transport to the customer [44].

Most companies decide to calculate the carbon footprint for their products, choosing the cradle to gate method. This method is more accurate, it has a lower risk of making a mistake, and allows us to examine all unit processes in the analyzed production system thoroughly. When calculating emissions from the cradle to the grave, many possible options should be considered, e.g., product use or disposal, and average values should be taken for situations that may or may not occur. It increases uncertainty and exposes the company to errors in calculating the carbon footprint of a product or technology. However, analyses in this scope are often carried out to assess the economic processes and comparative analyses [44].

Another environmental footprint which was investigated was an ecological footprint, i.e., the measure of the Earth’s biologically productive area necessary to produce 1 kg of the analyzed material. The ecological footprint is defined as the sum of the direct and indirect Earth surface utilization associated with the consumption of nuclear energy and CO_2_ emissions from the consumption of fossil fuels. The ecological footprint analysis allows the determination of human demand for natural resources in the biosphere. The ecological footprint estimates the amount of biologically productive land and water surface required to compensate for resources consumed for consumption, development, treatment of part of the waste and storage of other waste, as well as for the absorption of emissions resulting from the consumption of energy from fossil fuels and nuclear energy [45].

An ecological footprint is, by definition, an area of biologically productive surface, including land and water surface, the human population needed to meet consumption needs, and needed to assimilate emissions and absorb waste resulting from the use of energy from fossil fuels and nuclear energy. The ecological footprint is calculated for a specific period, usually for one year. The ecological footprint of the product is defined as the sum of the direct and indirect land surface used related to the consumption of nuclear energy, CO_2_ emissions from the consumption of fossil fuels and cement burning according to Equation (1):

EF = EF_direct_ + EF_CO2_ + EF_nuclear_ (1)

where:

- EF—ecological footprint;
- EF_direct_—the ecological footprint of direct land use in time; the following types of areas included were defined in the direct ecological footprint, which includes built-up areas, forests, arable fields, pastures, and the surface of water used for hydropower purposes;
- EF_CO2_—indirect land use in time; it is a biologically productive surface necessary to absorb CO_2_ emissions resulting from the energetic use of fossil fuels and cement production, through afforestation, i.e., introducing the forest to non-forest areas;
- EF_nuclear_—indirect land use in time; it is a biologically productive surface that is necessary to capture or absorb CO_2_ resulting from the nuclear energy use.

The EF ecological footprint is expressed in the following unit: m^2^·a (a—annually) [45].

Table 4 presents the values of equivalence factors for calculating individual components of the total value of the ecological footprint based on equivalence coefficients for different types of biologically productive areas. The equivalence factor (a type of weight) is used to transform the surface of a specific type of area (e.g., arable or forest area) into a universal unit of area biologically productive area. The values of land equivalence coefficients were determined on the assumption that one square meter of the land area corresponds to the average value of all bioproduct areas on Earth. The parameters presented in Table 4, equivalence factors for individual soil types, have different values depending on the degree of biological productivity of a given area, for example, arable fields have a higher coefficient value than pastures [45].

The direct ecological footprint is calculated using Equation (2):

EF_direct_ = Σ_a_A_a_ ×·eqF_a_ (2)

where:

- EF_direct_—ecological footprint of direct land use in time;
- A_a_—land development over time by using *a* type of land;
- eqF_a_—coefficient of equivalence of land use, type *a*.

The value of the next total component of the ecological footprint—indirect land use in time is calculated using Equation (3):

EF_CO2_ = M_CO2_ × (1 − F_CO2_)/S_CO2_ × eqF_f_ (3)

where:

- EF_CO2_—the ecological footprint of indirect land use—a biologically productive surface necessary to absorb CO_2_ emissions resulting from the energy use of fossil fuels and cement production, through afforestation, i.e., introducing forests to non-forest areas;
- M_CO2_—CO_2_ emissions attributed to the analyzed products;
- F_CO2_—CO_2_ fraction absorbed by oceans;
- S_CO2_—the degree of CO_2_ absorption by green plants;
- eqF_f_—forest equivalence coefficient.

Another element included in the total value of the ecological footprint is calculated using Equation (4):

EF_nuclear_ = E_nuclear_ × I_CO2_ × (1 − F_CO2_)/S_CO2_ × eqF_f_ (4)

where:

- EF_nuclear_—indirect land use—a biologically productive surface in time, necessary to absorb CO_2_ emissions arising from the use of nuclear energy;
- E_nuclear_—use of nuclear energy in the analyzed products;
- I_CO2_—CO_2_ emission intensity from fossil fuels;
- F_CO2_—CO_2_ fraction absorbed by oceans;
- S_CO2_—CO_2_ absorption by green plants;
- eqF_f_—forest equivalence coefficient.

The ecological footprint assessment provides decision-makers with information on the environmental impact of a single product or technology, as well as cities, regions, countries, continents, and even the world as a whole. The ecological footprint provides not only local but also global information. It can be used to assess the impact of human activities on the ecosystem. It contains integrated information on human activity, which directly or indirectly affects the development of areas diversified in terms of bioproductivity, including:

- agricultural production areas (livestock farming and crop production) and forest areas,
- areas necessary for CO_2_ absorption by green plants.

The assessment of the ecological footprint provides the opportunity to manage and monitor biological potential and to deal with it and also indicates its biophysical limitations.

**Figure 5:**
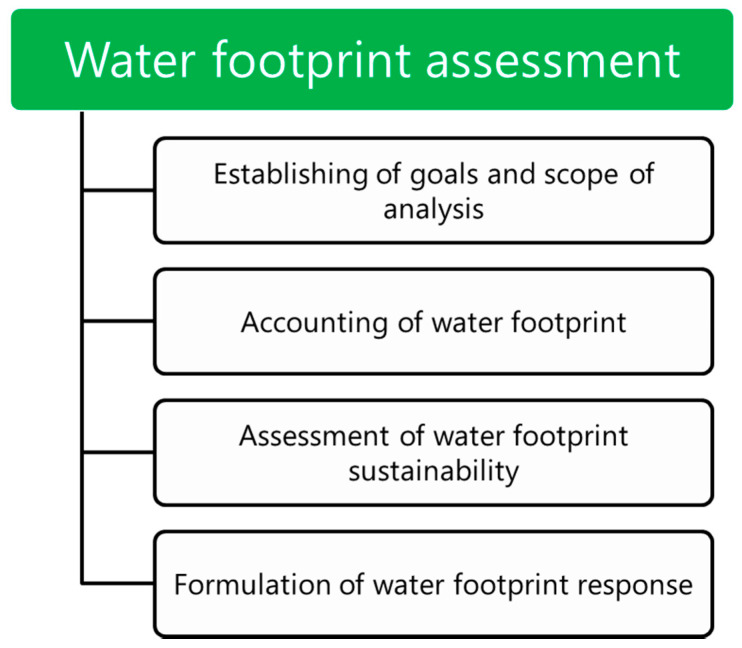
Schematic illustration of water footprint assessment.

The assessment of the water footprint is one of the latest methods of assessing the impact on the environment among the family of environmental footprints. The methodological basis for calculating the water footprint was developed by Hoekstra [46], and it was improved in the following years by him and his colleagues [47,48]. The water footprint is an indicator of freshwater consumption. It takes into account the direct consumption of water by the consumer and producer and its indirect consumption. Determining an individual water footprint is a modern method of measuring the amount of water consumed by an enterprise or consumer to meet their needs. The water footprint determines the direct and indirect demand for water of products and technologies. Human activity entails the consumption and pollution of water which is associated with irrigation, cooling, living and economic purposes, processing. These activities generate a water footprint. The water footprint presented in the analysis is the sum of the direct and indirect water footprints of the analyzed materials. A water footprint assessment is performed because it allows the determination of the dependencies between the economy and local water supplies. Governments and companies may calculate their impact on water supplies and limit water utilization, becoming more environmentally friendly. In the presented study, the main goal was to determine the water footprint of selected PP-based biocomposites. Evaluation of the water footprint for a EUR-pallet was performed according to the methodology proposed by Hoekstra et al. [47], schematically presented in Figure 5.

### 2.3. Input Data

According to the methodology mentioned above on environmental footprints calculations, it is necessary to gather the data necessary for assessment. For the presented work, data were collected from scientific publications as well as reports and databases including Ecoinvent database v 3.1. This information was also provided in our previous work [49]. The data available in the Ecoinvent database are developed based on technological data obtained from companies, industrial associations, and research institutes operating on the market. Then, obtained data are subjected to statistical treatment. The method and sources of data collection and the methodology of statistical processing of the obtained data are described in detail in the report: “Overview and methodology Data Quality Guideline for the Ecoinvent Database Version 3” [50].

Moreover, some assumptions have to be made due to the fact of some differences in particular production processes all over the world. Regarding polypropylene, there are different methods of production, based on various types of polymerization reactions. For analysis, it was assumed that:

- polymerization of propylene is performed with 95% yield;
- 25% of the production is based on suspension polymerization;
- 75% of the production is based on gas-phase polymerization;
- for both types of polymerization, 4 MJ of electric energy per kg of PP and 4 MJ of thermal energy is required.

Data used for environmental footprints assessment included obtaining and processing raw materials and transport and utilization of generated waste.

Information from the National Residential Efficiency Measures Database was used for the assessment of cotton fibers’ environmental footprints [51]. The database is operated by National Renewable Energy Laboratory (NREL)—a United States (US) federal laboratory performing analyses related to development, commercialization, and implementation of eco-saving technologies, often based on renewable energy and resources. In the presented work, data gathered from the NREL database were complemented with information collected from other reports [52].

For assessment of the environmental footprints of jute and kenaf fibers, data presented by the Natural Institute of Research on Jute and Allied Fibre Technology in India were applied [53]. In the analysis, information related to the whole life cycle was used, from crop cultivation to fiber production. Just as in the case of cotton fibers, data were complemented with information collected from other reports [54].

Data required for glass fibers were collected from leading European glass producers (26 production lines in 12 countries) and averaged. The assessment included obtaining and processing of raw materials including glass from recycling, transport, electricity usage, and waste management [55].

## 3. Results and Discussion

### 3.1. Environmental Footprints of EUR-Pallet

**Figure 6:**
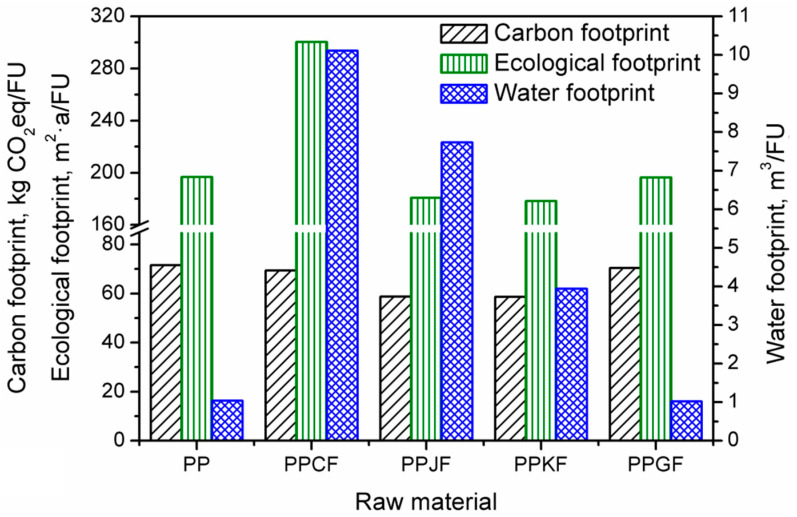
Values of the environmental footprints of EUR-pallet depending on analyzed composition variant.

The results for analyses of various material compositions of EUR-pallet can be seen in Figure 6. For comparison, the values of environmental footprints of a pallet prepared solely from PP and PP/glass fibers composite are also presented.

**Figure 7:**
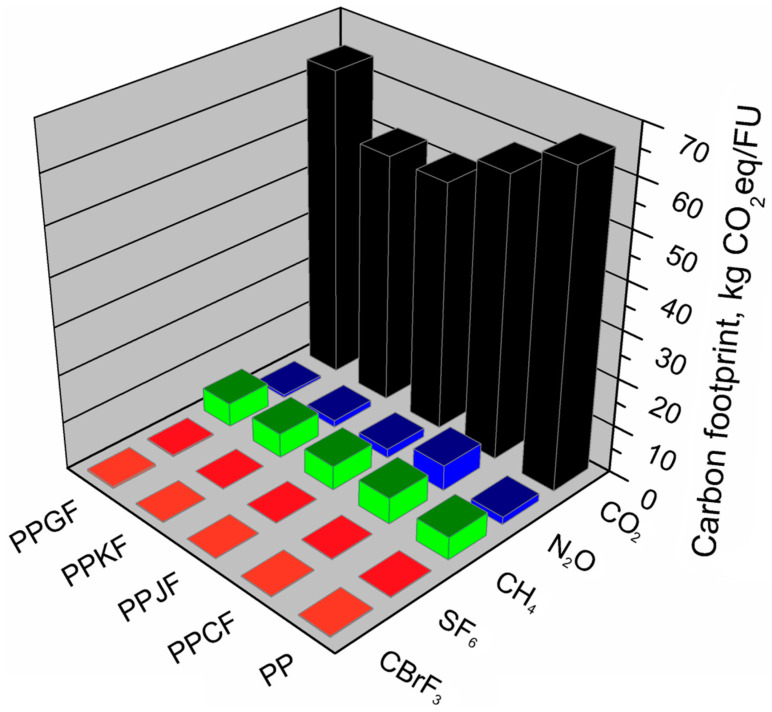
Shares of particular greenhouse gases in total carbon footprints of EUR-pallet depending on composition variant.

The analyses carried out allowed the assessment of the carbon footprint value of the model product in various material variants. It was indicated which greenhouse gases were emitted from individual production systems and what is their share in the total carbon footprint, expressed in CO_2_ equivalent. The carbon footprint of EUR-pallets manufactured by injection technology from various raw materials, in most of the material variants analyzed, was at a similar level, except for biocomposites reinforced with jute and kenaf fibers. In these variants, the carbon footprint was smaller than the other analyzed variants and took the values of 58.70 and 58.57 kg CO_2_eq/FU, respectively. The highest value of this indicator—71.53 kg CO_2_eq/FU—was observed for a pallet made of polypropylene. A similar carbon footprint had EUR-pallets made of glass fiber-reinforced polypropylene (PPGF)—70.35 kg CO_2_eq/FU and cotton fiber reinforced polypropylene (PPCF)—69.39 kg CO_2_eq/FU. As can be seen in Figure 7 presenting a share of particular GHGs in the total carbon footprint, carbon dioxide emissions showed the most significant impact. Their share in the total carbon footprint of the following material variants: PP, PPGF, slightly exceeded 90%. This was probably because polypropylene and glass fibers production belong to energy-consuming technological processes. Also, these processes are mainly based on non-renewable raw materials [56]. In the PPCF, PPJF, and PPKF material variants, the CO_2_ share in the carbon footprint was in the range of 85–88%. It has been shown that methane emission in the analyzed material variants constitutes from 7% to 8% of the carbon footprint. The highest methane emission occurs when using pure polypropylene while the lowest in the material variant in which polypropylene is reinforced with cotton fiber (PPCF). Nitrous oxide emission also occurs in the analyzed production systems, while its share in the total carbon footprint was insignificant, and in most of the analyzed variants, it constituted from 0.5% to 3%. The exception was the variant in which polypropylene was reinforced with cotton fiber (PPCF); then, the total emission of nitrous oxide from the production system represented 8% of the carbon footprint. The other two gases shown in the analyses, sulfur hexafluoride and bromotrifluoromethane, despite a very high greenhouse potential, accounted for very little of the carbon footprint of the analyzed EUR-pallets. In total, their share in the carbon footprint of individual variants did not exceed 0.5%.

**Figure 8:**
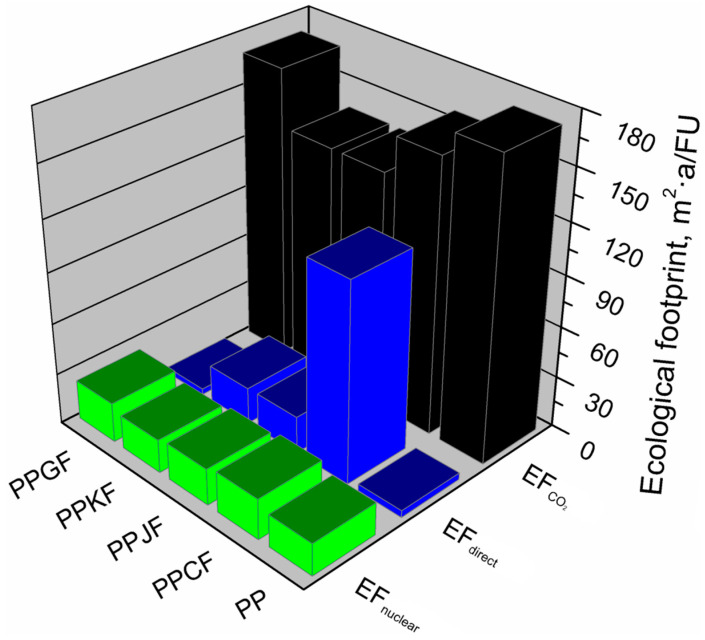
Shares of particular components in the total ecological footprints of EUR-pallet depending on composition variant.

The ecological footprint assessment results were expressed in square meters per year concerning the functional unit under analysis—m^2^·a/FU. Figure 8 presents the shares of the particular components in total ecological footprint for individual material variants used for the production of EUR-pallets.

Based on the results obtained, it was determined which factors and to what extent determine the size of the ecological footprint of individual material variants of the model product. The ecological footprint of the analyzed variants was for polypropylene (PP) 196.76 m^2^·a/FU, for cotton fiber-reinforced polypropylene (PPCF) 300.31 m^2^·a/FU, for glass fiber-reinforced polypropylene (PPGF) 196.39 m^2^·a/FU, for jute fiber-reinforced polypropylene (PPJF) 180.68 m^2^·a/FU, and kenaf fiber-reinforced polypropylene (PPKF) 178.37 m^2^·a/FU. To the greatest extent, the ecological footprint of all analyzed material variants, except for the PPCF composite, was affected by the indirect use of terrain over time, necessary for the absorption of CO_2_ emissions resulting from the energy use of fossil fuels (EF_CO2_). For material variants PP and PPGF, the share of this factor in the total value of the ecological footprint was from 80% to 88%. For PPJF and PPKF variants, this factor determines the value of the ecological footprint in 76% and 77%, respectively. The EF_CO2_ factor had the least impact on the ecological footprint value of cotton fiber-reinforced polypropylene (PPCF), contributing to 53% of the total ecological footprint value of this material variant. Such an effect was associated with significantly higher, compared to other variants, share of the direct use of a biologically productive surface (EF_direct_). In this material variant, this factor constitutes 39% of the ecological footprint value, while for the remaining compositions, it was in the range of 1–6% and 11–12%, respectively, for PP and PPGF and PPKF and PPJF. Based on the analysis of the results obtained, it can be concluded that higher values of EF_direct_ are characteristic for EUR-pallets, for the production of which raw materials of plant origin are used, as is the case with variants with natural fibers, cotton, jute, and kenaf.

Based on the analyses carried out, it was found that the factor—indirect land use necessary for the absorption of CO_2_ emissions resulting from the use of energy from nuclear fuels (EF_nuclear_), is similar in all material variants and accounts for 8% to 13%.

In Figure 6, there are also presented values of water footprint calculated for the five investigated composition variants of EUR-pallets. It can be seen that regarding the use of fresh water, the most environmentally friendly materials are obtained from non-renewable raw materials. The water footprint of neat polypropylene and its composite with glass fibers showed values of 1.02 and 1.04 m^3^/FU, respectively. At the same time, the replacement of 30 wt% of PP with kenaf, jute, and cotton fibers resulted in the increase of the water footprint to 3.94, 7.73, and 10.11 m^3^/FU, respectively. Such a significant rise of this indicator is related to the high water demand during the cultivation of crops, mainly for irrigation. Moreover, various protection aids, such as fertilizers and pesticides, are also characterized by relatively high water footprints. Agriculture is generally responsible for around 85% of the total use of surface and groundwater [57].

**Figure 9:**
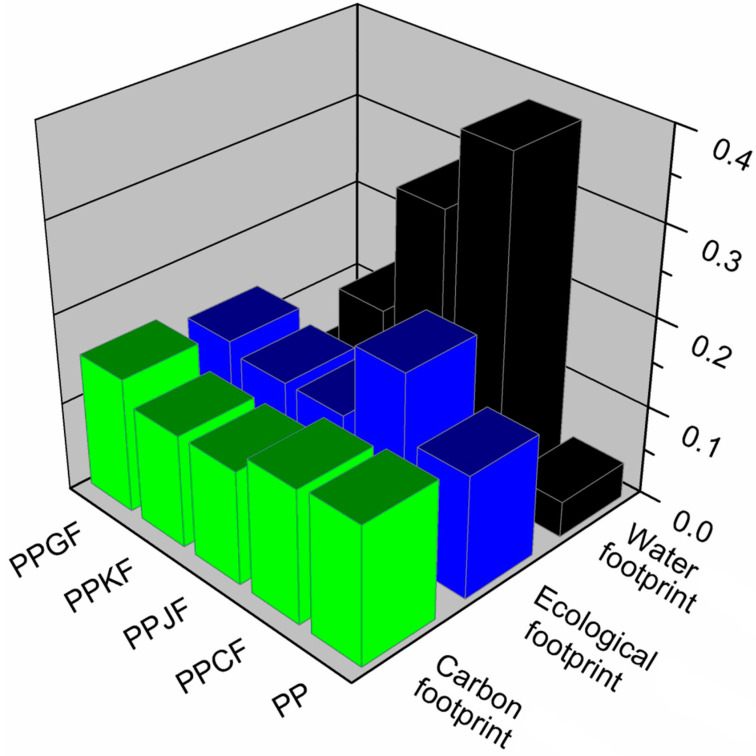
Normalized values of environmental footprints of EUR-pallet depending on composition variant.

The results obtained were normalized because each trace was expressed in a different unit to make a comparative analysis of the results obtained in assessing the environmental footprints of individual material variants of the EUR-pallets. It made it possible to compare environmental footprints as shown in Figure 9.

Based on the analysis of standardized results of environmental footprints of individual material variants of EUR-pallets, it was found that the use of natural fibers to make composites does not make the entirely sustainable materials. A raw material or semi-finished product of natural origin is not always environmentally friendly, as evidenced by the high value of their ecological or water footprint. Compared to glass and cotton fiber composites, biocomposites with jute and especially kenaf fibers are less harmful to the environment. The negative effect of cotton fibers, cultivated industrially on a large scale, is primarily associated with the irrigation of crops and the use of fertilizers and plant protection products that generate the size of the analyzed environmental footprints. Of the fibers analyzed, the use of cotton fiber was the most unfavorable regarding a negative impact on the environment.

**Figure 10:**
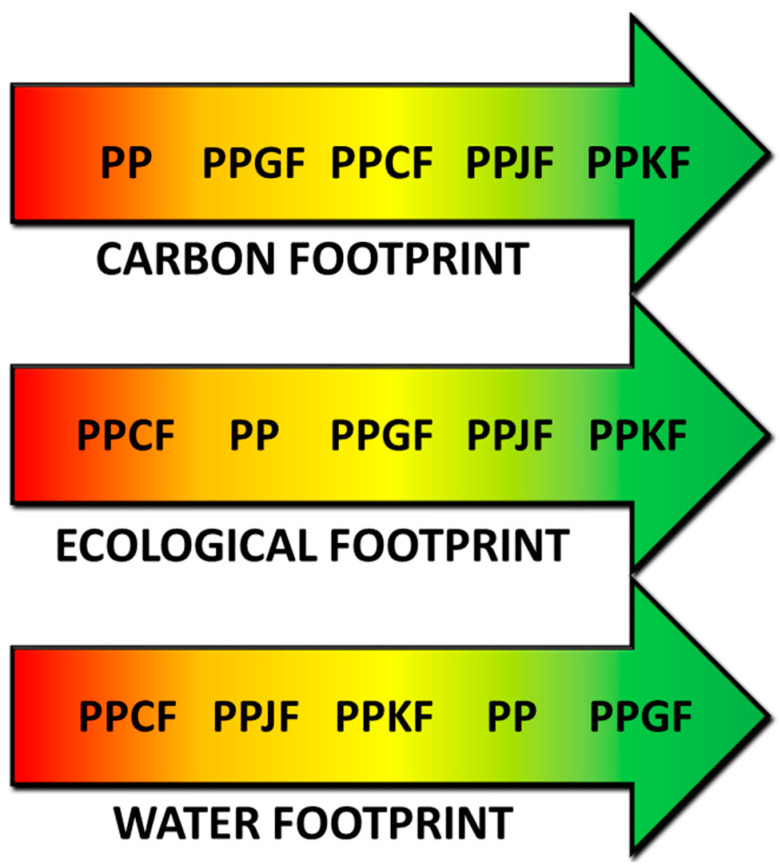
Diagram showing recommended (green) and non-recommended (red) composition variants of EUR-pallet depending on environmental footprint.

Based on the obtained results of assessments of individual environmental footprints, a diagram of recommended materials for an example product was prepared concerning individual environmental footprints (Figure 10).

Individual material variants were ranked, starting with those not recommended due to the high values of environmental footprints (area marked in red) to the most recommended variants due to the lowest environmental footprints (area marked with green). This form of presenting the results of analyses of compared products or technologies provides decision makers with information on the environmental impact in a clear and easy to interpret manner. This diagram can also be helpful at the product design stage. Where among the available pool of materials with similar processing and functional properties, suitable for a given application, one can select and recommend a material with the least negative impact on the environment for a given environmental footprint.

### 3.2. Environmental Footprint of Applied Raw Materials

Generally, the results of carbon footprint assessment are in line with the common understanding of the environmental friendliness, since the recommended materials are obtained by partially replacing petroleum-based polymers with natural fibers. Nevertheless, values of ecological and water footprints are significantly different and indicate that natural fiber composites are rather not recommended. For clarification and a better understanding of the origin of the presented results, detailed analyses of environmental footprints are presented for all analyzed raw materials.

#### 3.2.1. Polypropylene

Table 5 presents the polypropylene production system components that directly and indirectly affect its environmental footprints. Based on the obtained results, it has been shown that during polypropylene manufacturing, the most significant impact on the environment is shown by the production of monomer and the use of electricity. The first factor is mainly affecting carbon and ecological footprints (over 66%), while for water footprint, the most aggravating is the second one (60%). Together, independently of footprint, their share exceeds 84% of the total impact. The impact of heat applied for the manufacturing of polypropylene, irrespectively of its source, shows significantly lower environmental impacts.

**Figure 11:**
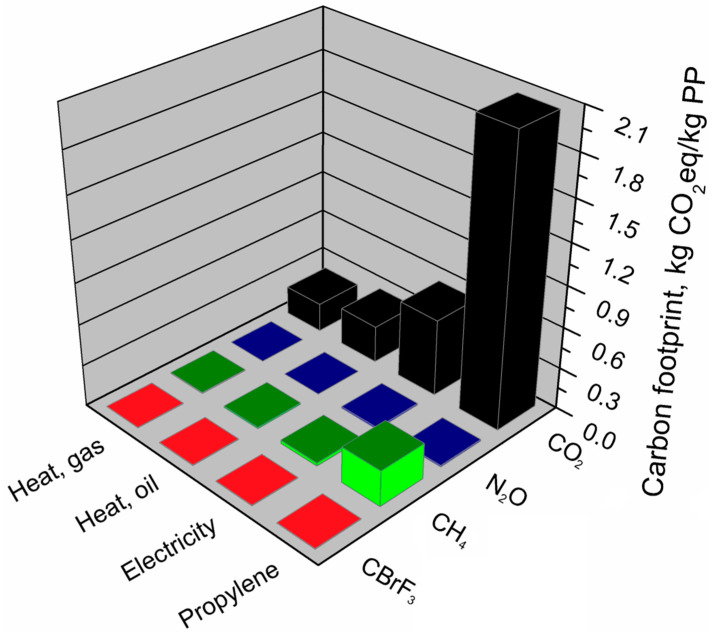
Shares of individual GHG emissions in the main components of the polypropylene production system.

For a more detailed analysis, Figure 11 shows the volume of individual greenhouse gas emissions from the main components of the polypropylene production system.

It was found that the most significant impact on the carbon footprint of polypropylene had carbon dioxide emission during the production of monomer-polymerization propylene and indirect CO_2_ emissions related to the production of electricity used in the polypropylene production system. The CO_2_ emissions generated over 90% of the carbon footprint of the polypropylene production system. In the case of nitrous oxide, indirect emissions were associated with electricity generation, while the highest emission of nitrous oxide occurs in the production of monomer-polymerization propylene. This component is also responsible for the highest emission of methane and bromotrifluoromethane in the analyzed production system. It was shown that among the analyzed processes, the lowest greenhouse gas emissions in the polypropylene production system come from heat generation processes during the combustion of heating oil and refinery gas.

Of the three elements of the ecological footprint (i.e., EF_direct_, EF_CO2_, EF_nuclear_), only indirect land development associated with the use of the biologically productive surface necessary for CO_2_ absorption, resulting from the use of energy from fossil fuels, affects the ecological footprint of the polypropylene production system. Therefore, shares of E_CO2_ correspond to the total ecological footprint of polypropylene.

#### 3.2.2. Cotton Fibers

Table 6 presents the components of the cotton fiber production system that directly and indirectly affect the value of the environmental footprints. Contrary to PP, it can be seen that for cotton fibers, the share of particular components differs significantly considering various footprints. Nevertheless, there are four components with the most significant impact on the environmental footprints of the cotton fiber production system—production of fibers, application of fertilizers, irrigation, and technological operations related to crop cultivation. They stand for 86%, 94%, and 99.6%, respectively, of carbon, ecological, and water footprints. Such a high share of irrigation in the total value of the water footprint of cotton fibers was discussed in our previous work and the works of other research groups [49,58,59].

**Figure 12:**
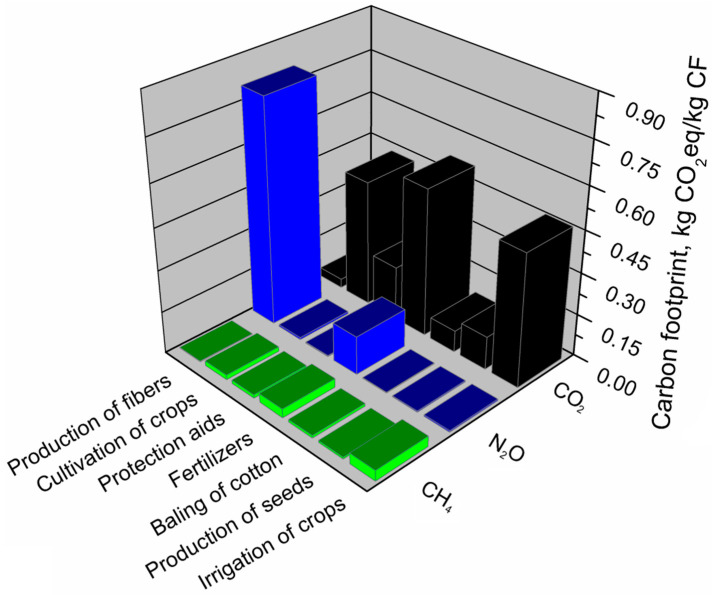
Shares of individual GHGs emissions in the main components of the cotton fibers production system.

Growing cotton on a massive scale is associated with a heavy burden on the environment. To protect the harvest against pests and diseases and to enhance production yield, chemical preparations are used to affect the environmental footprints of the cotton production system significantly. The use of fertilizers has an unusually high impact on the carbon footprint of the cotton fiber production system. Their impact is more than three times greater than in the case of pesticides. A high value of the carbon footprint generated by irrigation and mechanized cotton cultivation, including operations related to plowing, harrowing, mulching, sowing or harvesting, results from large areas characteristic for cotton plantations [60]. Figure 12 shows all greenhouse gas emissions calculated for the cotton fiber production system.

Based on the analyses carried out, it was determined which greenhouse gases and the extent of the carbon footprint of each component in the cotton fiber production system. Carbon dioxide is a greenhouse gas that determines the carbon footprint of a cotton fiber production system. Carbon dioxide emissions are primarily affected by the use of fertilizers, irrigation, crops related to cotton (plowing, harrowing, mulching, sowing, harvesting), and the use of pesticides. Total CO_2_ emissions account for over 60% of all greenhouse gas emissions from the cotton fiber production system. Nitrogen oxide also has a significant impact on the carbon footprint value of the analyzed fibers, whose total emission in the cotton fiber production system calculated as equivalent carbon dioxide is over 30%. This gas is emitted mainly during the production of fibers. The use of fertilizers also affects the emission of nitrous oxide. The share of methane emissions in the cotton fiber production system is small, but it does not exceed 4%. The irrigation of crops mainly causes the methane emission in the cotton fiber production system, the use of fertilizers, and technological processes related to the automation of cotton cultivation (plowing, harrowing, harvesting).

**Figure 13:**
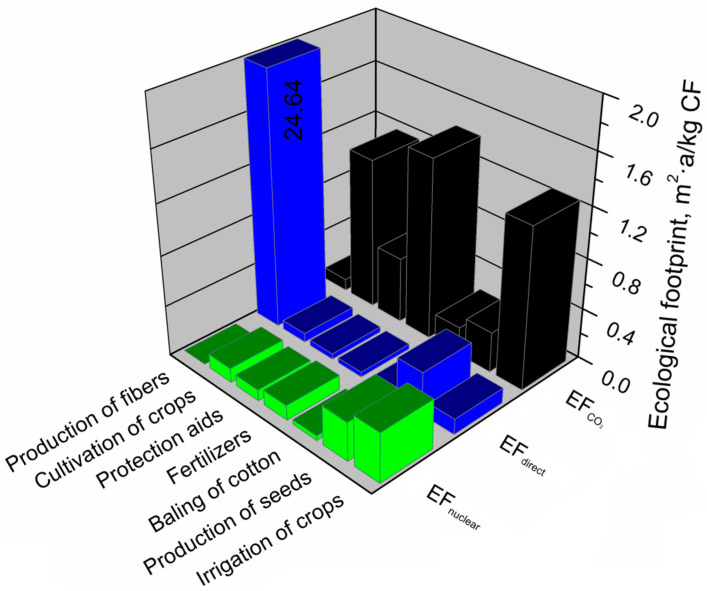
Shares of particular elements of the ecological footprint in the main components of the cotton fibers production system.

The most significant impact on the ecological footprint of the cotton fiber production system was the stage of obtaining fibers, where the share in the ecological footprint of this system was 79%. The least impact on the ecological footprint of the cotton fiber production system had cotton baling operations and processes related to obtaining cotton grain and the use of plant protection products. Figure 13 presents a detailed analysis of the main aspects of the cotton fiber production system generating an ecological footprint and its components (EF_direct_, EF_CO2_, EF_nuclear_), determining this footprint.

As mentioned above, the ecological footprint of cotton fibers was most affected by the fiber production stage. It is over ten times larger than for the other components of the production system. Over 99% of the ecological footprint value of this component is generated by direct land use (EF_direct_). In the case of cotton production, it was probably due to the vast area of a cotton plantation. The value of the ecological footprint of the component—the production of cotton grain—is determined to the greatest extent by the EF_direct_ factor. For the share of other factors (EF_CO2_, EF_nuclear_) in the component, cottonseed production was at a similar level. Of the other components of the cotton fiber production system, the most substantial impact on the size of the ecological footprint is primarily irrigation of crops, use of fertilizers, operations related to cotton cultivation (plowing, harrowing, mulching, sowing, harvesting), and the use of pesticides. Among these components, the primary determinant of the ecological footprint is the indirect land use necessary to absorb CO_2_ emissions as a result of energy use from fossil fuels (EF_CO2_).

#### 3.2.3. Jute Fibers

The assessment of the environmental footprints of jute fibers allowed us to determine the impact of the main components of the jute fiber production system which is presented in Table 7. Significantly, the highest impact is presented by the cultivation of crops, however, in the case of carbon footprint. Also, the application of fertilizers and pesticides showed a 23% share. Generally, the cultivation stage is the most mechanized, and machines and devices powered by electricity are used to process the fibers. Moreover, it includes the irrigation of crops which results in the very high share of this stage in the total water footprint of jute production. The lowest environmental impact on jute production is shown by transport, which is often much less mechanized and made without the use of specialized equipment resulting in low environmental burdens [53].

**Figure 14:**
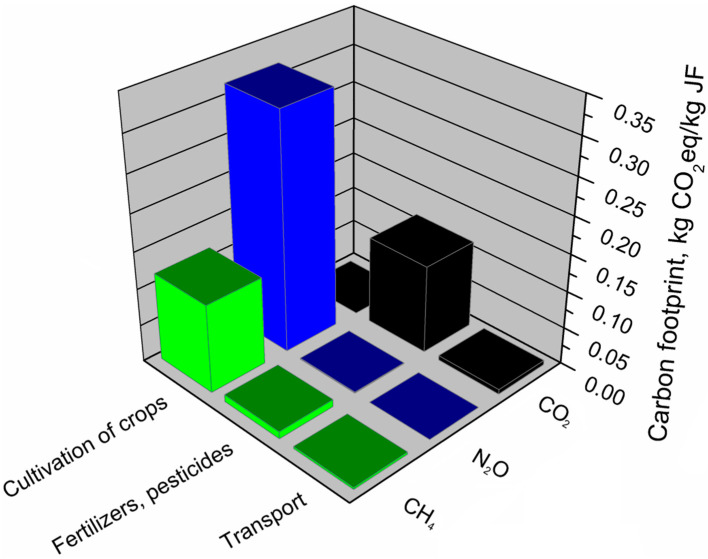
Shares of individual GHG emissions in the main components of the jute fibers production system.

Figure 14 presents data on emissions of all greenhouse gases from the jute fiber production system; it indicates which greenhouse gases and to what extent they affect the carbon footprint of individual components in the analyzed production system.

The conducted analyses showed that the emission of carbon dioxide did not determine the value of the carbon footprint of jute fibers. Even though the stage of jute growing and fiber production generated 75% of the carbon footprint value of the jute fiber production system, it did not emit CO_2_ that was absorbed by plants during their growing season. The carbon footprint of fibers is determined mainly by the emission of nitrous oxide and methane, which generated almost 78% of CO_2_ equivalent emissions in the entire jute fiber production system. Referring the obtained results to the greenhouse potential of the analyzed gases (Table 3), only 1.08 g of nitrous oxide emitted from the production system generates 321 g of CO_2_ equivalent, while in the case of methane, the emission of 117 g of CO_2_ corresponds to 4.68 g of methane. The highest CO_2_ emissions in the jute fiber production system are caused by the use of plant protection products and mineral fertilizers. Carbon dioxide is also emitted during transport in the jute fiber production system.

**Figure 15:**
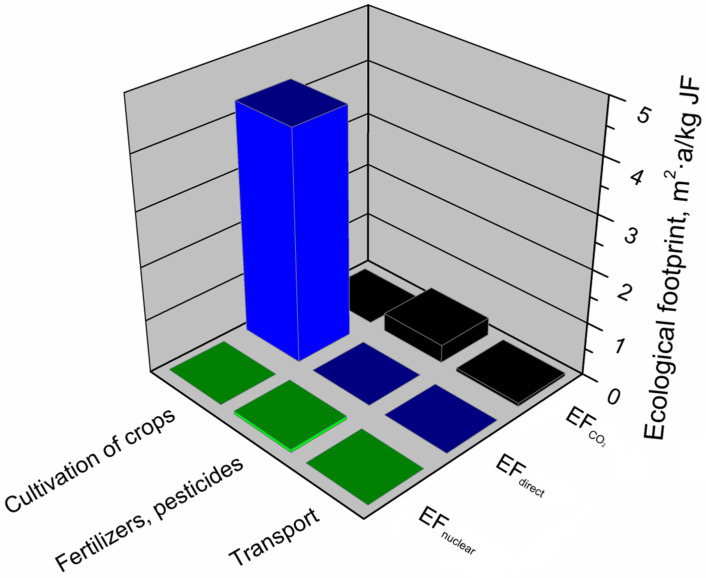
Shares of particular elements of the ecological footprint in the main components of the jute fibers production system.

As mentioned above, the ecological footprint of the jute fiber production system was primarily influenced by the stage of jute growing and fiber production, representing 91% of the ecological footprint of this system. This was due to the use of biologically productive areas for jute growing. Figure 15 indicates the jute fiber production system’s main components generating an ecological footprint and the factors (EF_direct_, EF_CO2_, EF_nuclear_) determining this footprint.

The analyses showed that the value of the ecological footprint of the jute fiber production system was mainly determined by jute cultivation and fiber production. This component was only affected by the direct use of biologically productive cultivation space (EF_direct_). The use of fertilizers and pesticides had a much lower impact on the ecological footprint of jute fibers and was mainly determined by the indirect use of land over time for the absorption of CO_2_ emissions arising from the energy use of energy from fossil fuels (EF_CO2_). The value of this component was also slightly affected by the indirect land use necessary to absorb CO_2_ emissions resulting from nuclear fuel (EF_nuclear_). The total value of the ecological footprint of the jute fiber production system was least affected (1%) by transport, where the dominant factor was indirect use in terrain, necessary to absorb CO_2_ emissions resulting from the energy use of energy from fossil fuels (EF_CO2_).

#### 3.2.4. Kenaf Fibers

Table 8 presents the impact of individual components of the kenaf fiber production system on its total environmental footprints. It can be seen that the shares of particular components are relatively similar for jute fibers. Significantly the highest shares were noted for the cultivation of crops, and again, it is associated with the use of machines, devices, and simultaneously, electricity. Moreover, a significant amount of water and the direct use of biologically productive areas are required to cultivate crops. The use of plant protection products and fertilizers had a significant impact on the environmental footprints of kenaf fibers and the most crucial stage in their production in chemical plants.

**Figure 16:**
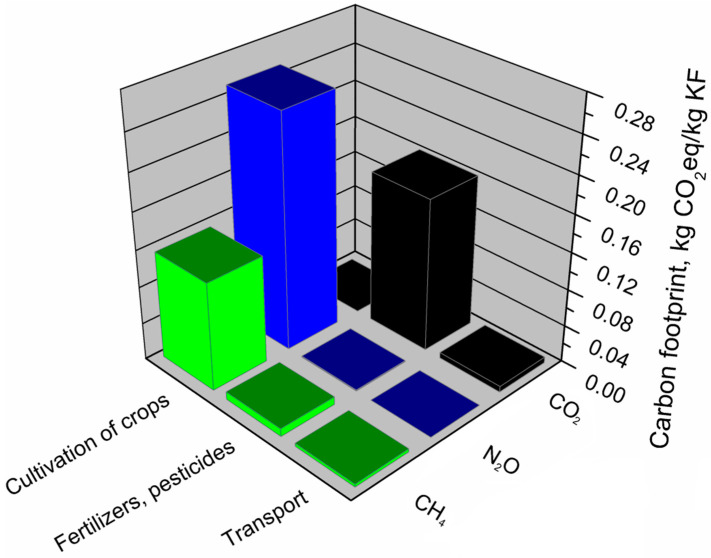
Shares of individual GHG emissions in the main components of the kenaf fibers production system.

Figure 16 shows the greenhouse gas emissions calculated for the kenaf fiber production system. The analyzes showed that the carbon footprint of kenaf fibers, similar to jute fibers, was determined by the emission of nitrous oxide and methane. Although the stage of kenaf growing and obtaining fibers generated 67% of the carbon footprint of the kenaf fiber production system, it did not emit CO_2_, which was absorbed by plants during the growing season. The carbon footprint of kenaf fibers was mainly influenced by the emission of nitrous oxide and methane. Relating the obtained results to the greenhouse potential of the analyzed gases (Table 3), only 0.85 g of N_2_O emitted from the kenaf fiber production system generated almost 256 g of CO_2_ equivalent, while 5 g of methane was responsible for 125 g of CO_2_ equivalent. In the case of CO_2_, the most substantial emissions from the kenaf fiber production system were caused by the use of plant protection products and mineral fertilizers. Of the greenhouse gases analyzed, CO_2_ was also emitted to the greatest extent during transport and cultivation in the kenaf fiber production system.

**Figure 17:**
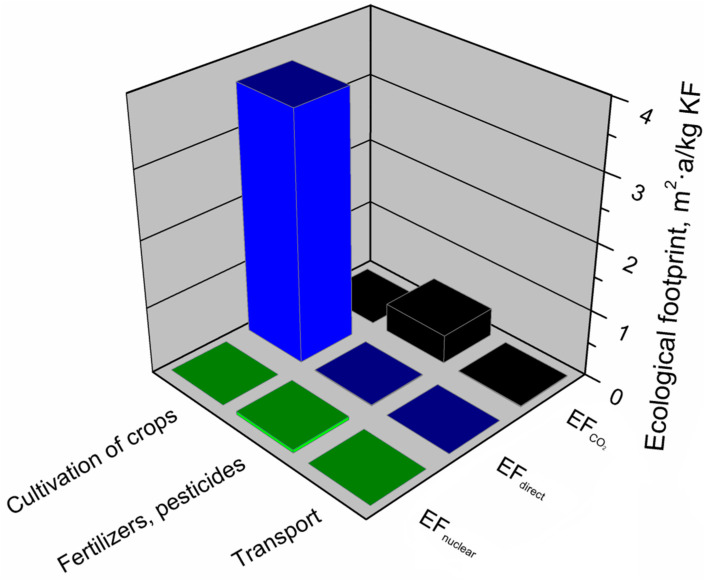
Shares of particular elements of the ecological footprint in the main components of the kenaf fibers production system.

Figure 17 presents the main components of the kenaf fiber production system generating an ecological footprint and the factors (EF_direct_, EF_CO2_, EF_nuclear_) determining this footprint. Analyses have shown that the value of kenaf fiber production’s ecological footprint was mainly influenced by kenaf cultivation and fiber production, which is directly affected only by the use of biologically productive terrain (EF_direct_) over time. The use of fertilizers and pesticides have a much lower impact on the ecological footprint of kenaf fibers and is mainly determined by the indirect use of terrain overtime to absorb CO_2_ emissions resulting from the energetic use of fossil fuels (EF_CO2_). The value of this component is also slightly influenced by indirect land use necessary to absorb CO_2_ emissions resulting from the use of nuclear energy (EF_nuclear_). The overall value of the ecological footprint of the kenaf fiber production system is least affected by transport, where indirect time use dominates, which is necessary to absorb CO_2_ emissions from the use of fossil energy (EF_CO2_).

#### 3.2.5. Glass Fibers

Table 9 presents the main components of the glass fiber production system that affect its environmental footprints. It can be seen that for glass fibers, the shares of individual components differred significantly between footprints. The carbon footprint of glass fibers is primarily determined by the consumption of natural gas and electricity in high-temperature glass production and processing. Total greenhouse gas emissions related to energy use (heat and electricity) in the glass fiber production system accounts for 83% of the carbon footprint. In the analyzed production system, the carbon footprint is slightly affected by the stages of obtaining raw materials and producing fibers. The lowest greenhouse gas emissions come from transport and processes related to wastewater and waste management. Regarding the ecological footprint, total energy use (heat and electricity) show a similar share (84%) as for the carbon footprint. However, individual shares of electricity and natural gas are reversed. A different situation is observed for the water footprint of glass fibers production. The most significant impact on water footprint (~74%) is noted for the production and use of applied raw materials, e.g., silica, aluminum oxide, boric acid, clays, fluorite, lime, and other additives and modifiers. The significant impact of the total water footprint was also observed for the use of electricity, similarly as in the case of other indicators. For all of the analyzed footprints, the lowest shares (~1%) were observed for transport and sewage management.

**Figure 18:**
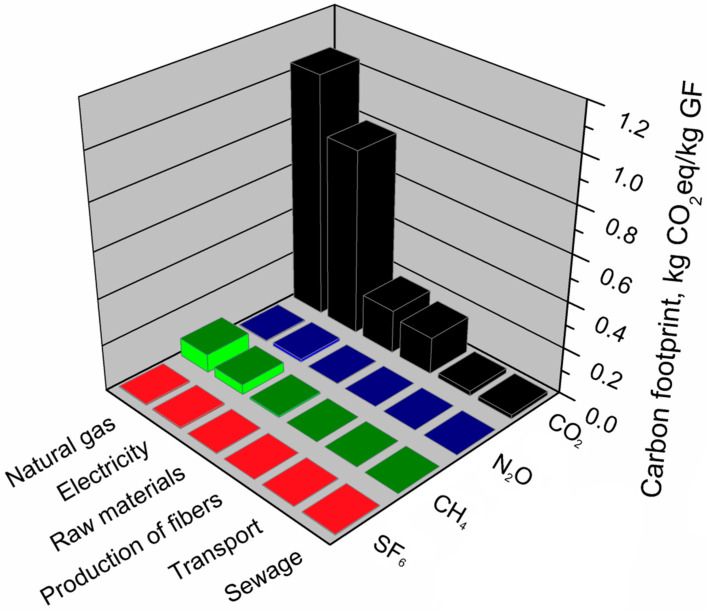
Shares of individual GHG emissions in the main components of the glass fibers production system.

Figure 18 presents data on emissions of all greenhouse gases related to the glass fiber production system. It was indicated which greenhouse gases and to what extent affected the carbon footprint of individual components in the analyzed production system. The carbon footprint of the glass fiber production system is determined by carbon dioxide emissions which account for over 94% of total greenhouse gas emissions. The most substantial CO_2_ emissions in the analyzed system were caused by the energy consumption of glass processing and production processes and the use of raw materials necessary for the production of glass fibers. Methane emissions in terms of equivalent carbon dioxide generated more than 5% of total greenhouse gas emissions. The most significant methane emission was associated with the use of heat energy from gas and electricity. The other two gases-nitrous oxide and sulfur hexafluoride generated less than 1% of greenhouse gas emissions in terms of carbon dioxide equivalents. Emission of sulfur hexafluoride nitrate oxide was mainly associated with the consumption of electricity and natural gas and with the use of raw materials used in the glass fiber production system.

**Figure 19:**
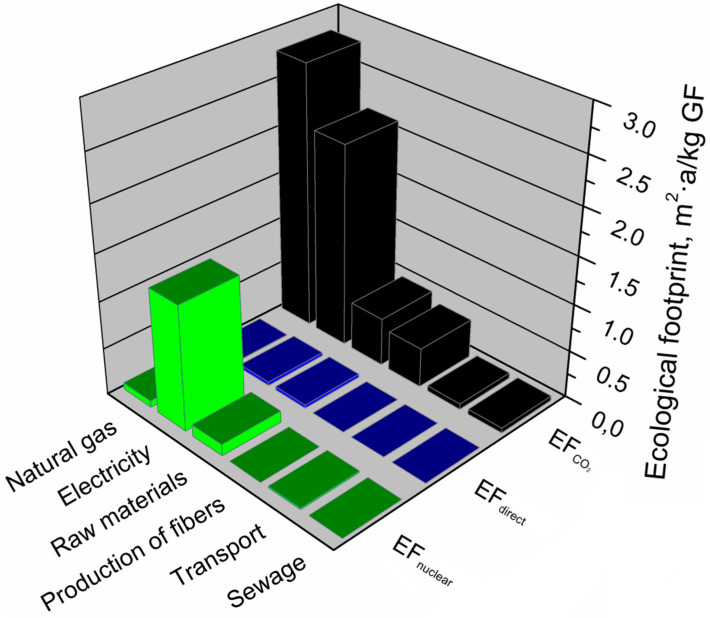
Shares of particular elements of the ecological footprint in the main components of the glass fibers production system.

Figure 19 presents the results of the analysis broken down into individual factors included in the ecological footprint of the glass fiber production system. The figure indicates the main components of the glass fiber production system that generate an ecological footprint and shows which factors (EF_direct_, EF_CO2_, EF_nuclear_) and to what extent determine the ecological footprint of glass fibers.

The ecological footprint of the glass fiber production system was determined by the indirect use of the biologically productive surface, necessary to absorb CO_2_ emissions resulting from the energetic use of energy from fossil fuels (EF_CO2_), which accounts for over 78% of the ecological footprint of glass fibers. This factor was dominant in all components of the glass fiber production system presented in the analysis, except for the stage of using electricity, where almost 40% of the ecological footprint value was generated by the indirect use of biologically productive surface necessary to absorb CO_2_ emissions resulting from the use of nuclear energy (EF_nuclear_). A slight impact of indirect land use for CO_2_ absorption arising from the use of nuclear energy was also demonstrated in components—raw materials and natural gas. It has been shown that among all the main components of the glass fiber production system for ecological footprint components—raw materials and electricity. Additionally, EF_direct_ has little effect on land use in time.

#### 3.2.6. Summary

**Figure 20:**
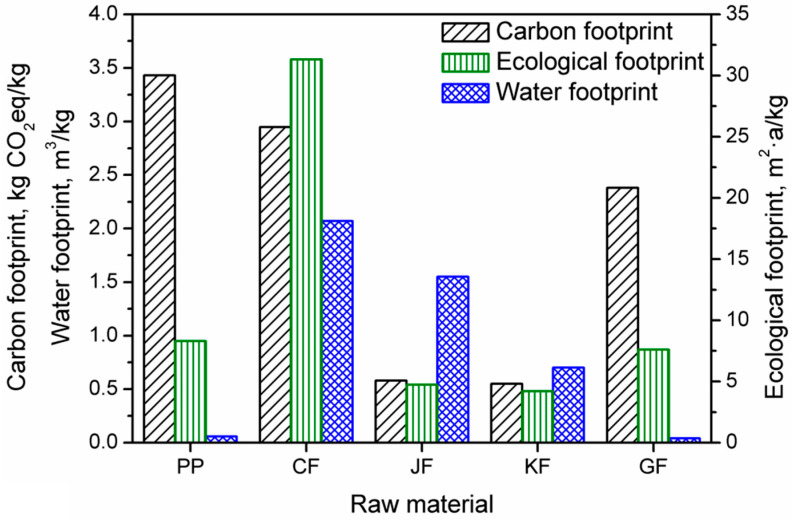
Values of the environmental footprints of analyzed raw materials.

Figure 20 presents the results of the environmental footprint assessment of the analyzed materials. Based on the analysis of the obtained results, it can be seen that all analyzed fibers show lower values of a carbon footprint than the polypropylene matrix (3.43 kg CO_2_eq/kg). Among fibers, the highest carbon footprint was noted for cotton fibers and amounts to 2.95 kg CO_2_eq/kg, thus exceeding the value of the carbon footprint of glass fibers, for which it is 2.38 kg CO_2_eq/kg. The lowest carbon footprint among the fibers analyzed was found for jute and kenaf fibers, 0.58 kg CO_2_eq/kg and 0.55 kg CO_2_eq/kg, respectively.

Jute and kenaf fibers also showed relatively low ecological footprint values, 4.73 and 4.21 m^2^·a/kg, respectively. Cotton fibers have the most significant ecological footprint of all the analyzed materials, 31.32 m^2^·a/kg. For polypropylene, the ecological footprint value was 8.31 m^2^·a/kg.

Regarding the water footprint, the highest values were noted for natural fibers, 0.70, 1.55, and 2.07 m^3^/kg for kenaf fibers, jute fibers, and cotton fibers, respectively. As mentioned above, it is related to the cultivation and irrigation of crops used in the production of fibers. For glass fibers, the water footprint was significantly lower—0.041 m^3^/kg; therefore, considering only the water footprint, glass fibers can be considered a lower environmental burden than natural fibers. For PP, the value of the water footprint equaled 0.059 m^3^/kg.

A detailed analysis of the components of their production systems that generate the carbon footprint was performed for individual materials. Table 10 summarizes the emissions of all greenhouse gases shown in the analyses. The share of individual greenhouse gases in the carbon footprint of the analyzed materials is indicated.

Table 11 summarizes the ecological footprint assessment results of the analyzed materials and indicates the share of individual factors (EF_direct_, EF_CO2_, EF_nuclear_) in their ecological footprint. The summary of the obtained results of greenhouse gas emissions has led to the conclusion that for conventionally applied materials, polypropylene, and glass fibers, the carbon footprint is by far mainly determined by CO_2_ emissions, which has shares that exceed 90%. Such an effect is associated with the energy consumption of these materials. It was demonstrated by the analyzes of individual components of production processes. For natural fibers, mainly jute and kenaf, there was an entirely different relationship. The emission of nitrous oxide primarily influences the carbon footprint of these materials. It was found that this was due to the use of plant protection products and fertilizers during production (plant vegetation period) as well as to fiber extraction and extraction processes from biomass.

It can be seen that for natural fibers (cotton, jute, and kenaf), that the size of the ecological footprint is determined by the direct development of biologically productive areas (EF_direct_) used for plant cultivation from which natural fibers are obtained. However, in the case of cotton fibers, the size of the ecological footprint over 16% was also affected by indirect development of (green) areas necessary to absorb CO_2_ emissions resulting from the use of energy from fossil fuels (EF_CO2_). For jute fibers, this factor generated 7.8% of the ecological footprint, and for kenaf fibers, this effect was over 10%. This involved intensive cotton cultivation on large mechanized farms, in contrast to jute and kenaf fibers, which are grown in tropical countries, mainly on smaller farms and using agricultural machinery and equipment on a much smaller scale than is the case for cotton. The ecological footprint of glass fibers was almost 100% affected by the indirect use of biologically productive areas (green areas) to absorb CO_2_ resulting from the energy use of fossil fuels (78.9%) and to absorb CO_2_ emissions resulting from the use of nuclear energy (20.3%). This was mainly due to the nature of the processes related to the acquisition and preparation of raw materials (mainly mineral) for the production of glass and high energy consuming processing processes aimed at obtaining glass fibers. In the case of polypropylene, only one factor had an impact on the value of its ecological footprint—use during biologically productive areas necessary for the absorption of CO_2_ emissions resulting from the energetic use of energy from fossil fuels (EF_CO2_) which is mainly associated with the use of energy during the acquisition of fossil raw materials for the production of propene and its polymerization as well as in the processing and modification of polypropylene.

## 4. Conclusions

The results of the presented study show a comparative analysis of the environmental footprints of polypropylene and its composites filled with glass fibers as well as cotton, jute, and kenaf fibers based on a standardized EUR-pallet case study. Plastic EUR-pallets are commonly used all over the world in different branches of industry. Generally, the incorporation of natural fibers into polymer matrices is commonly considered as a pro-ecological solution. Considering the carbon footprint of material, such a statement is definitely true. Composites containing 30 wt% of cotton, jute, and kenaf fibers showed a 3%, 18%, and 18% lower value of this indicator comparing to neat polypropylene. Differences among particular fibers were associated with the technology related to cultivation and collecting of crops. Cultivation of cotton is strongly mechanized, including operations related to plowing, harrowing, mulching, sowing or harvesting, while the same operations for jute or kenaf fibers are often performed manually. Moreover, due to the high demand for cotton, significantly higher amounts of fertilizers and protection aids are used, which affects the ecological footprint of cotton. As a result, polypropylene/cotton fibers composites showed over 52% higher value of this indicator comparing to neat polypropylene, while the use of jute and kenaf fibers enabled its 8.2% and 9.4% reduction. Regarding the water footprint, the incorporation of natural fibers definitely cannot be considered an environmentally friendly solution. The EUR-pallet made with composites containing 30 wt% of cotton, jute, and kenaf fibers showed a water footprint of 3.94, 7.73, and 10.11 m^3^, while for neat polypropylene its value was 1.02 m^3^. Comparing to natural fibers, the introduction of glass fibers resulted in only slight changes in the environmental indicators. Carbon and ecological footprints were reduced by 1.6% and 0.2%, respectively, while the water footprint increased by 2.0%.

Generally, in a broad view, regarding all environmental aspects, the use of natural fibers in the manufacturing of polymer composites, definitely cannot be impartially considered as an environmentally friendly solution. Nevertheless, the main burden—water footprint—is strongly related to the origin of natural fibers. Therefore, it can be reduced by proper selection and use of fibers, e.g., by the use of various waste materials, which could be applied as a source of natural fillers in the manufacturing of polymer composites. Such an approach should definitely be considered as a future direction of research work related to composites containing natural fillers.

To sum up, we believe that the approach based on the assessment of multiple environmental footprints may be a handy tool for the plastics industry. It enables extensive and comprehensive analysis of materials and production processes and their impact on the environment. Such analysis could definitely be very helpful for the engineering of processes and materials with the lowest possible impact on the natural environment. Based on the presented data, future directions of research in the area of natural fiber composites and wood polymer composites should include the following issues:

- Application of waste materials or by-products, especially those, which are currently unmanaged, could significantly reduce the environmental impact of polymer composites. Depending on the adopted model, environmental burdens of recycled materials are at least partly allocated to the primary process, and sometimes these materials are even burden-free;
- Among potential candidates for lignocellulosic fillers could be mentioned various types of biomass or wastes from the food industry, which are treated as municipal solid waste. Often applied incineration usually does not enable the utilization of the full energetic potential of the material, and results in the generation of significant amounts of exhaust gases, increasing the environmental footprints;
- Comparing to incineration, more beneficial is mechanical recycling allowing the use of lignocellulosic materials directly in the new application. The most promising are materials whose utilization do not require additional treatment such as drying, which requires a substantial amount of energy, increasing the environmental impacts of the process;
- Assessment of the environmental impacts of products or processes should not be limited only to basic indicators, mainly related to the use of fossil fuels and global warming potential. Instead, it should include comprehensive analysis associated with the use of natural resources, e.g., water, whose scarcity is considered as a growing problem. Such an approach should be emphasized, nowadays, when the number of processes and products based on natural and renewable raw materials is increasing.

## Figures and Tables

**Table 1:** Advantages and drawbacks of water, ecological, and carbon footprints.

	Carbon Footprint	Ecological Footprint	Water Footprint
Advantages	enables a comprehensive assessment of greenhouse gas emissions; complies with economic and environmental reporting standards; includes the emissions of all gases showing greenhouse potential; the emission data obtained are comparable and available to most countries.	enables comparative analysis of human demand for renewable resources and expresses human needs related to the absorption of emissions and waste concerning the “supply” of nature; enables aggregated assessment of various anthropogenic impacts on the ecosystem; is easy to communicate and understand, and contains a strong message about the protection of natural goods; identifies human impact on Earth’s ecosystem and biodiversity and measures its negative impact.	expresses the need for water resources on a micro (product, technology, enterprise) and macro (region, country, planet) scale; extends traditional water intake measurements, shows the relationship between local consumption and the global distribution of freshwater; integrates water use and pollution in the production chain.
Drawbacks	focuses on only one category of environmental impact, omits other equally critical environmental aspects; cannot follow the full range of human needs for the environment; additional impact assessment models are needed to analyze the impact of climate change at national and subnational levels.	does not cover all aspects of sustainable development or all environmental issues; especially those for which there is no renewable potential; shows which factors can lead to degradation of natural capital (e.g., reduced land quality or reduced biodiversity) but does not forecast such degradation; is geographically ambiguous.	it only tracks human demand for freshwater; relies on local data, often not available or difficult to obtain; calculations for gray water are based on assumptions and estimations; depending on local water purity standards; calculations for gray water may be different for the same products or technologies in different regions.

**Table 2:** Composition variants of analyzed EUR-pallet.

Component	Variant				
	PP	PPCF	PPJF	PPKF	PPGF
	Content, wt%				
Polypropylene	100	70	70	70	90
Cotton fibers	-	30	-	-	-
Jute fibers	-	-	30	-	-
Kenaf fibers	-	-	-	30	-
Glass fibers	-	-	-	-	10

**Table 3:** Values of global warming potential for particular greenhouse gases.

Greenhouse Gases		Gas Global Warming Potential, g CO_2_eq/kg
Carbon dioxide	CO_2_	1
Methane	CH_4_	25
1,1-Difluoroethane	C_2_H_4_F_2_	120
Nitrous oxide	N_2_O	298
1,1,1,2-Tetrafluoroethane	CH_2_FCF_3_	1300
Heptafluoropropane	C_3_HF_7_	3500
1,1,1-Trifluoroethane	C_2_H_3_F_3_	4300
Bromotrifluoromethane	CBrF_3_	7140
Octafluoropropane	C_3_F_8_	8600
Octafluorocyclobutane	C_4_F_8_	10,000
Hexafluoroethane	C_2_F_6_	11,900
Trifluoromethane	CHF_3_	12,000
Sulfur hexafluoride	SF_6_	22,800

**Table 4:** Values of equivalence factors for calculating individual components of the ecological footprint.

Parameter	Abbreviation	Unit	Value
Equivalence factor for forests	eqF_f_	-	1.4
Equivalence factor for built-up lands	eqF_b_	-	2.2
Equivalence factor for arable lands	eqF_c_	-	2.2
Equivalence factor for hydroenergetic water	eqF_h_	-	1.0
Equivalence factor for pastures	eqF_p_	-	0.5
Equivalence factor for fishing grounds	eqF_m_	-	0.4
Fraction of CO_2_ absorbed by oceans	F_CO2_	-	0.3
Degree of CO_2_ absorption by plants	S_CO2_	kg CO_2_·m^−2^·a^−1^	0.4
Intensity of CO_2_ emission from fossil fuels	I_CO2_	kg CO_2_·MJ^–1^	0.07

**Table 5:** Environmental footprints of particular components of the polypropylene production system.

Component of the Production System	Carbon Footprint		Ecological Footprint		Water Footprint	
	kg CO_2_eq/kg PP	%	m^2^·a/kg PP	%	m^3^/kg PP	%
Electricity	0.60	18	1.49	18	0.0356	60
Propylene	2.33	68	5.52	66	0.0219	37
Heat, oil	0.28	8	0.73	9	0.0010	2
Heat, gas	0.22	6	0.56	7	0.0005	1
Total	3.43	100	8.31	100	0.0590	100

**Table 6:** Environmental footprints of particular components of the cotton fibers production system.

Component of the Production System	Carbon Footprint		Ecological Footprint		Water Footprint	
	kg CO_2_eq/kg CF	%	m^2^·a/kg CF	%	m^3^/kg CF	%
Production of fibers	0.84	28	24.73	79	0.0000	0.0
Cultivation of crops	0.47	16	1.37	4	0.0022	0.1
Protection aids	0.21	7	0.65	2	0.0015	0.1
Fertilizers	0.70	24	1.57	5	0.0153	0.7
Baling of cotton	0.09	3	0.26	1	0.0004	0.0
Production of seeds	0.13	4	0.96	3	0.0051	0.2
Irrigation of crops	0.52	18	1.78	6	2.0455	98.8
Total	2.95	100	31.32	100	2.0700	100

**Table 7:** Environmental footprints of particular components of the jute fibers production system.

Component of the Production System	Carbon Footprint		Ecological Footprint		Water Footprint	
	kg CO_2_eq/kg JF	%	m^2^·a/kg JF	%	m^3^/kg JF	%
Cultivation of crops	0.44	75	4.30	91	1.5476	99.8
Fertilizers, pesticides	0.13	23	0.38	8	0.0024	0.2
Transport	0.01	2	0.05	1	0.0000	0.0
Total	0.58	100	4.73	100	1.5500	100

**Table 8:** Environmental footprints of particular components of the kenaf fibers production system.

Component of the Production System	Carbon Footprint		Ecological Footprint		Water Footprint	
	kg CO_2_eq/kg KF	%	m^2^·a/kg KF	%	m^3^/kg KF	%
Cultivation of crops	0.37	67	3.71	88	0.6984	99.8
Fertilizers, pesticides	0.17	31	0.49	12	0.0014	0.2
Transport	0.01	2	0.01	0	0.0000	0.0
Total	0.55	100	4.21	100	0.6999	100

**Table 9:** Environmental footprints of particular components of the glass fibers production system.

Component of the Production System	Carbon Footprint		Ecological Footprint		Water Footprint	
	kg CO_2_eq/kg GF	%	m^2^·a/kg GF	%	m^3^/kg GF	%
Production of fibers	0.16	7	0.41	5	0.0000	0.0
Electricity	0.86	36	3.53	46	0.0094	23.0
Natural gas	1.13	47	2.88	38	0.0009	2.2
Raw materials	0.19	8	0.67	9	0.0303	73.9
Transport	0.02	1	0.07	1	0.0003	0.7
Sewage	0.02	1	0.05	1	0.0001	0.3
Total	2.38	100	7.60	100	0.0411	100

**Table 10:** Emissions of particular greenhouse gases during the production of analyzed materials.

Material	Greenhouse Gas				
	CO_2_, %	N_2_O, %	CH_4_, %	CBrF_3_, %	SF_6_, %
PP	90.4	0.6	8.9	0.1	-
GF	94.3	0.4	5.2	-	0.1
CF	63.7	32.7	3.6	-	-
JF	21.7	56.3	22.0	-	-
KF	30.7	46.5	22.8	-	-

**Table 11:** Shares of individual components in total values of the ecological footprint of analyzed materials.

Material	Individual Component of the Ecological Footprint		
	EF_direct_, %	EF_nuclear_, %	EF_CO2_, %
PP	-	-	100
GF	0.8	20.3	78.9
CF	80.6	3.3	16.1
JF	91.2	1.0	7.8
KF	88.3	1.1	10.6
